# 

**Published:** 1879-10

**Authors:** 


WHOLE NUMBER, CCXIX. OCTOBER, 1879. NUMBER 3, VOLUME XIX.

### THE BUFFALO Medical and Surgical Journal.

INDEX MEDICUS.
S.G.O.L INDEXED

EDITORS:

BUFFALO:
PUBLISHED BY THE MEDICAL JOURNAL ASSOCIATION.

**Read the Notice of this Journal among the Advertisements.**

Entered at the Post-Office at Buffalo, N. Y., as Second-Class Mail Matter.

